# Reason as Our Guide

**DOI:** 10.1371/journal.pbio.0020116

**Published:** 2004-03-05

**Authors:** Elizabeth Blackburn, Janet Rowley

## Abstract

Two scientist members of the President's Council on Bioethics express their concerns about two recently issued reports by the Council in which the science is presented incompletely and myths are perpetuated

We are two of the scientist members of the President's Council on Bioethics. In late 2001, we were invited by the President of the United States to serve on this Council. The Bioethics Council was appointed by the President to “monitor stem-cell research, to recommend appropriate guidelines and regulations, and to consider all of the medical and ethical ramifications of biomedical innovation…. This council will keep us apprised of new developments and give our nation a forum to continue to discuss and evaluate these important issues.”

This was a difficult invitation to accept. On the one hand, the President's views on the use of human embryonic stem cell research and somatic cell nuclear transfer techniques were well-known and in conflict with our own beliefs about the costs and benefits of the use of progressive technologies to advance biomedical research. On the other hand, we were grateful that the President, despite his views in opposition to these therapies, was willing to invite serious biomedical scientists to help formulate advice to him—and ultimately to contribute to the development of national policy—on these critically important advances.

We knew that on this originally 18-member (but for most of the past two years a 17-member) Council, as scientists we would be in the minority in our belief of the good to be gained through these and other areas of biomedical research. We were also aware that some others on the Council had strong opposing views. Thus, it was only with the assurances of the Council chairman, Leon Kass of the University of Chicago, and of the President of the United States himself that we were persuaded that our voices would be heard and integrated into the statements of the Council. Furthermore, we felt, and continue to feel, that bioethical issues are important not only to all biologists, but also to society at large, and thus especially worthy of engaging debate and discussion.

Two recently issued reports of the Council, “Beyond Therapy: Biotechnology and the Pursuit of Happiness” (http://bioethics.gov/reports/beyondtherapy/index.html) and “Monitoring Stem Cell Research” (http://bioethics.gov/reports/stemcell/index.html), are therefore of deep concern to us. We discuss them in turn below.

## Concerns about the “Beyond Therapy” Report

The “Beyond Therapy” report deals with issues of direct concern for every thoughtful person. However, in the interests of setting straight the record of our views, as Council members and scientists, on the content of this report and for a proper assessment of the scientific content of the “Beyond Therapy” report, we feel it is important to point out aspects of the report for which we had requested revisions and for which those requests were declined.

In the discussions of preimplantation genetic diagnosis, the specter of designer babies is raised by implying that selecting embryos for intelligence and other traits, such as temperament is a possibility. Scientifically, this simply is highly unlikely and indeed may not even be feasible. While such scientific unlikelihood is mentioned in passing in the report, it is easy to take away from the report the feeling that such genetic manipulation will happen and is even imminent.

The report also claims that “the underlying impulse driving age-retardation research is, at least implicitly, limitless, the equivalent of a desire for immortality.” Furthermore, the title of Chapter 4 of the report, “Ageless Bodies,” implies that immortality is the goal of this research, despite all reliable scientific evidence to the contrary. Such a title is not consistent with the knowledge, stated in that chapter, that there is no scientific basis for immortality and implies that, by seeking to maintain and extend “youth,” research into aging, including stem cell research, is predominantly to serve vanity. Also, without presenting scientific or reliable evidence, the report presents the opinion that research into prolonging healthy life may result in a lifetime obsession with immortality. Hence, this chapter in the report falls short of explaining the serious challenge of preventing and curing age-related disease to extend health—very different from attempting immortality.

The same chapter offers a sensational quote from a researcher that “the real goal [of aging research] is to keep people alive forever.” The request that quotes from researchers more representative of the biomedical research community also be included was declined. This leads to a misleading misrepresentation of the motivation of reputable researchers in the field of aging.

In suggesting that slowing biological aging may increase the disjunction between “social aging” (the age at which children are exposed to “adult” images and concepts) and “biological aging” (expected lifespan), only one view, a conservative one, of the supposed “best” way to raise children is presented. The report also suggests, with no clear reasoning behind it, that longer lives will somehow undermine human determination to contribute as much as one can during a lifetime. Despite requests for inclusion of material that would allow for a balanced treatment of these topics, the report minimized discussion of potential positive aspects of slowing biological aging, such as prolonged good health.

Finally, the report repeatedly emphasizes a “profound and mysterious” link between longevity and fertility, thereby leaving the reader with the distinct but erroneous impression that anything done to extend healthy life will be traded for decreased fertility, despite the fact that current scientific literature, which was made available for inclusion in the report, shows a *lack* of any necessary *mechanistic* linkage of the two.

## Concerns about the “Monitoring Stem Cell Research” Report

With respect to the “Monitoring Stem Cell Research” report, we feel that some facts that would help the public and scientists better assess the content of the report were not brought out clearly or were omitted entirely. First, from the published scientific literature in peer-reviewed journals on stem cells, a major message can be distilled: namely, the vast difference that currently exists in our understanding of, and the potential utility of, embryonic versus adult stem cells as sources of material for research and clinical purposes. In brief, human stem cells have been isolated from a variety of embryonic, fetal, and adult tissue sources. However, enormous differences exist in purity, properties, data reproducibility, and understanding of cells from these different sources. Much of our ignorance is related to the relative paucity of funding for research using embryonic stem cells.

Years of rigorous and careful research in animal models have documented that embryonic stem cells have great utility for scientific studies. This work has also rigorously and reproducibly established the great plasticity of these cells and supports the opinion that human embryonic stem cells possess the greatest broadest potential and promise for clinical applications. As well as therapeutic uses, important potential applications include studies of embryonic stem cells bearing complex genotypes susceptible to poorly understood common human diseases and testing and screening drug efficacy.

The report does not make clear that the best-characterized adult stem cells are hematopoietic stem cells. Currently, major difficulties and inadequate understanding exist with most other types of adult stem cells reported to date. In addition, many experiments suggesting that adult stem cells have broad plasticity may be incorrectly interpreted owing to an error caused by an experimental artifact of cell fusion present in some unknown proportion of the experiments. Research on some of the reported adult stem cell preparations may conceivably in the future demonstrate that they, too, like hematopoietic stem cells, can also be prospectively identified, “single cell cloned,” expanded considerably by growth in vitro with retention of normal chromosome structure and number, and preserved by freezing and storage at low temperatures. But it should be strongly cautioned that this has not been done for most adult stem cell preparations, and, even if possible, it is not clear that any of the just-mentioned procedures will be accomplished in the near future, owing to the technically very demanding nature of such experiments.

We feel it is important to emphasize a point that the report mentioned, that the reported isolation and properties of multipotent adult progenitor cells (MAPCs) must be reproduced in additional laboratories for any reliable interpretation of the results reported with these cells. After considerable effort, this has still not been achieved. Thus, in the reported results, the possible significance of the reported isolation and properties of human MAPCs is left unclear, as is their potential as a source of stem cells for clinical purposes. Hence, a strong overall caution is that many of the reports on the properties of cells differentiated from adult stem cell preparations are to date preliminary and incomplete. If results with any isolated and characterized adult stem cells are validated, it will then be very important to compare their properties—and those of any more differentiated cells that can be derived from them—with other stem cell sources, such as the well-characterized hematopoietic stem cells, and with human embryonic stem cell preparations.

Two major considerations argue strongly for non-commercial, federal, peer-reviewed funding to be made available for this work. The first is the sustained effort this work will require. The second is the importance of reliable and unbiased design of experiments and of open, public availability of the complete findings.

## Reasons for Our Concern

In being concerned about the content of these reports, neither of which makes any recommendations for legislative or policy actions, are we worrying too much? We think not. Indeed, already, sadly as a result of the way the sections on aging research in the report were written, the myth that longevity has an inevitable tradeoff of diminished fertility is now gaining a further foothold: witness the January 26, 2004, issue of the *The New Republic*. In it, an article about this report of the Council falls right into the trap: it states, “But changes come with longer life. Worms and mice that are altered for extended lifespans become sterile, or barely reproduce.” The public is done a disservice when science is presented incompletely; myths are then perpetuated.

This is but one example of the dangers that three of the Council members who are scientists (the two of us along with Michael Gazzaniga of Dartmouth College) pointed out, in a Commentary within the edition of the “Beyond Therapy” report published by the Dana Foundation in November 2003. In that Commentary, we stated that “Our concern … is that, moving forward, the debate carry on with all of the scientific evidence—or as much as such a widespread public discussion can include—and take care not to leave an erroneous impression as to the nature of the potential problems at hand.” We ended the Commentary by saying “We urge both good reading and *critical* reading!” (our italics).

These reports had as their premise the aim of neutrality in the scientific analysis of the issues addressed. But our concern is that some of their contents, as in the few examples outlined above, may have ended up distorting the potential of biomedical research and the motivation of some of its researchers. Continuing discussions will form the basis for future decisions on these topics; keeping such discussion open and balanced is of paramount importance.

##

**Box 1. President's Council on Bioethics**

The President's Council on Bioethics was created on November 28, 2001. Its mission includes: to “advise the President on bioethical issues that may emerge as a consequence of advances in biomedical science and technology. In connection with its advisory role, the mission of the Council includes the following functions:

1. to undertake fundamental inquiry into the human and moral significance of developments in biomedical and behavioral science and technology;
2. to explore specific ethical and policy questions related to these developments;
3. to provide a forum for a national discussion of bioethical issues;
4. to facilitate a greater understanding of bioethical issues; and
5. to explore possibilities for useful international collaboration on bioethical issues.”

From Executive Order 13237

George W. Bush

The White House, November 28, 2001

Federal Register date: November 30, 2001

Federal Register page: 66 FR 59851

The members of the President's Council on Bioethics at the time these reports were written included Leon R. Kass, M.D., Ph.D. (Chair), American Enterprise Institute; Elizabeth H. Blackburn, Ph.D.*, University of California, San Francisco; Rebecca S. Dresser, J.D., M.S., Washington University School of Law; Daniel W. Foster, M.D., University of Texas, Southwestern Medical School; Francis Fukuyama, Ph.D., Johns Hopkins University; Michael S. Gazzaniga, Ph.D., Dartmouth College; Robert P. George, J.D., D.Phil., Princeton University; Mary Ann Glendon, J.D., M.Comp.L., Harvard University; Alfonso Gómez-Lobo, Dr. Phil., Georgetown University; William B. Hurlbut, M.D., Stanford University; Charles Krauthammer, M.D., syndicated columnist; William F. May, Ph.D.*, Southern Methodist University; Paul McHugh, M.D., Johns Hopkins Hospital; Gilbert C. Meilaender, Ph.D., Valparaiso University; Janet D. Rowley, M.D., University of Chicago; Michael J. Sandel, D.Phil., Harvard University; and James Q. Wilson, Ph.D., University of California, Los Angeles.

* These members had their Council terms terminated by White House directive on February 27, 2004.

## Abbreviation

MAPC: multipotent adult progenitor cell
